# Difference between CD25^+^Tregs and Helios^+^Tregs in a murine model of allergic rhinitis

**DOI:** 10.1016/j.bjorl.2019.12.001

**Published:** 2020-01-11

**Authors:** Yun-Xiu Wang, Zhao-Wei Gu, Zhi-Wei Cao

**Affiliations:** a China Medical University, Shengjing Hospital, Department of Medical Insurance, Shenyang, China; b China Medical University, Shengjing Hospital, Department of Otolaryngology Head and Neck Surgery, Shenyang, China

**Keywords:** Allergic rhinitis, CD25^+^Treg, Helios^+^Treg

## Abstract

**Introduction:**

Regulatory T or Treg cells, balance the peripheral immune response to allergens in allergic rhinitis. Traditionally, Treg (CD25^+^ Treg) is identified by the coexpression of Foxp3 and CD25, but this strategy does not represent the true inhibitory function of Treg cells. Helios has been thought of as novel marker of activated Tregs, with an important inhibitory function. Consequently, Helios was proposed as a marker of Treg. Recent articles have shown that Foxp3 and Helios co-expression (Helios^+^Tregs) is an important functional stage of Treg.

**Objective:**

To compare the prevalence of CD25^+^Tregs and Helios^+^Tregs using a mouse model of allergic rhinitis.

**Methods:**

Twenty mice were randomized into two groups. The test group comprised 10 allergic rhinitis model mice exposed to ovalbumin; the control group was exposed to saline. The fractions of CD25^+^Tregs, Helios^+^Tregs, Helios^+^CD25^+^, and Helios^+^Foxp3^+^CD25^+^Tregs present in the two groups were determined using flow cytometry.

**Results:**

CD25^+^Tregs and Helios^+^Tregs were less abundant in the spleen and nasal mucosa cells of the allergic rhinitis model compared with the control. We also observed fewer Helios^+^Tregs than CD25^+^Tregs in nasal mucosa and splenic cells of both control and test groups. Moreover, we observed fewer Helios^+^Foxp3^+^, Helios^+^CD25^+^, and Helios^+^Foxp3^+^CD25^+^ Tregs in the nasal mucosa in the allergic rhinitis model. Helios was expressed the most in CD4^+^ CD25^+^Foxp3^+^ T-cells, followed by CD4^+^ CD25^−^Foxp3^−^ T-cells. Approximately 75% of CD25^+^Tregs were Helios^+^ in spleens of allergic rhinitis and control mice.

**Conclusion:**

This is the first report of the proportions of Helios^+^Tregs in nasal mucosa and spleens of allergic rhinitis mice. Gating true inhibitory Tregs with the coexpression of Foxp3 and Helios might be more useful than relying on the expression of CD25. This study provides a new insight for Treg studies of allergic rhinitis, and the potential utility of the marker as a therapeutic target.

## Introduction

In immune system, regulatory T (Treg) cells balance the peripheral immune response to allergens.^1^ They inhibit the effector T cells in a Th1 or Th2 phenotype inflammatory reaction and serve an important function in allergic rhinitis (AR). The techniques used to evaluate Treg cell activity have been modified. Early studies measured the expression of CD25 to quantify Treg cells.^2^ The inhibitory role of Treg cells is mediated via Foxp3 expression, which is considered the most specific marker.^3^, ^4^ Traditionally, Treg (CD25^+^Treg) is identified through the coexpression of Foxp3 and CD25. However, Foxp3 and CD25 are transiently upregulated in some other conditions,^5^, ^6^, ^7^, ^8^ so their coexpression may not truly represent suppressive Tregs. Some believe that thymic-derived natural CD4^+^Foxp3^+^Treg cells specifically express the Ikaros transcription family member Helios.^9^ Recent articles have shown that coexpression of Foxp3 and Helios is a critical functional phase of Treg, and it has recently been hypothesized that Helios may be an activator of Treg cells,^10^ and that Helios^+^FoxP3^+^Treg (Helios^+^Treg) may actually be more inhibitory than Helios^−^FoxP3^+^Treg.^11^, ^12^ There has been no related research on AR.

This report compared CD25^+^Treg and Helios^+^Treg cells using flow cytometry in murine AR model.

## Methods

### The mouse model

Female 8 week-old BALB/c mice were exposed to a 12 h light and dark cycle with access to food and water ad libitum. There was no Ovalbumin (OVA) in the food. This experiment was approved by Shengjing Hospital Ethics Committee (2018PS96K), China Medical University.

### Murine model of AR induced by OVA

The animals were split into two groups of 10 mice each. On days 0, 7 and 14, OVA group were immunized with 100 μg OVA and 2 mg aluminum hydroxide via intraperitoneal injection. Then, 100 μg of OVA was introduced nasally daily for 7 consecutive days starting on day 21. The control group was challenged with saline.

### Evaluation of AR symptoms

Sneezing and nasal rubbing were recorded on day 27, 15 min after the final intranasal introduction of OVA. The average of the counts was analyzed statistically between the OVA group and control group.

### Nasal mucosal tissue evaluation

All animals were euthanized and decapitated 2 h after the final OVA challenge. The heads of the five animals in each group were fixed with 4% paraformaldehyde. The samples were decalcified in 10% ethylenediaminetetraacetic acid. Paraffin-embedded samples were sectioned at 4 μm thicknesses and stained with hematoxylin and eosin to visualize eosinophils.

### Isolation of nasal mucosa and spleen cells

Nasal mucosa and spleen cells were isolated from the remaining mice, crushed in a glass grinder with RPMI 1640, and then filtered using a cell strainer. After centrifuging the cellular suspension, the proportions of Treg cells in the pellets were determined.

### Enumeration of Treg cell proportions using flow cytometry

Cells were stained with Fluorescein Isothiocyanate (FITC)-labeled anti-CD4 Abs and PerCP-Cy5.5-labeled anti-CD25 Abs (BD Biosciences), and then fixed and permeabilized using a fix/perm solution. The cells were then incubated with PE-labeled anti-Foxp3 Abs and Allophycocyanin (APC)-labeled anti-Helios (BD Biosciences). The data were obtained using a BD FACSCanto II flow cytometer.

### Statistical analysis

The results are expressed as means ± SEM. GraphPad Prism was used for the statistical analyses. Differences between the two groups were analyzed using the Mann–Whitney U-test; *p* < 0.05 indicated statistical significance.

## Results

### Symptom score in the AR murine model

**Figure 1:**
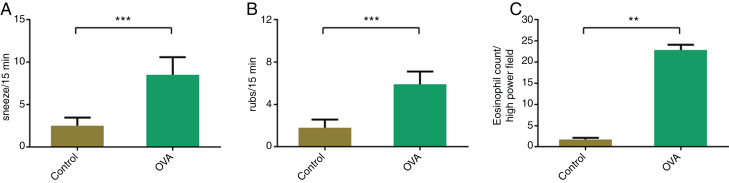
Comparison of nasal symptoms and eosinophilic infiltration between the Ovalbumin (OVA) and control groups: (A) sneezing, (B) nasal rubbing, and (C) eosinophil counts. **p < 0.01, ***p < 0.001.

On day 27 of the experiment, nasal rubbing and sneezing were counted for 15 min after the final introduction of OVA nasally (Fig. 1A and B). The OVA-induced mice sneezed significantly more frequently than the control animals (8.5 ± 2.1 vs. 2.5 ± 1.0, *p* < 0.05). The frequency of rubbing was also significantly higher in the OVA group (5.9 ± 1.2 vs. 1.8 ± 0.8, *p* < 0.05).

### Greater eosinophil infiltration into nasal mucosa of the AR mice

The infiltration of eosinophils into the nasal mucosa was evaluated after H&E staining and was significantly greater in the OVA group (*p* < 0.05) (Fig. 1C).

### Reduced CD25+Tregs in the spleen and nasal mucosa of the AR model

**Figure 2:**
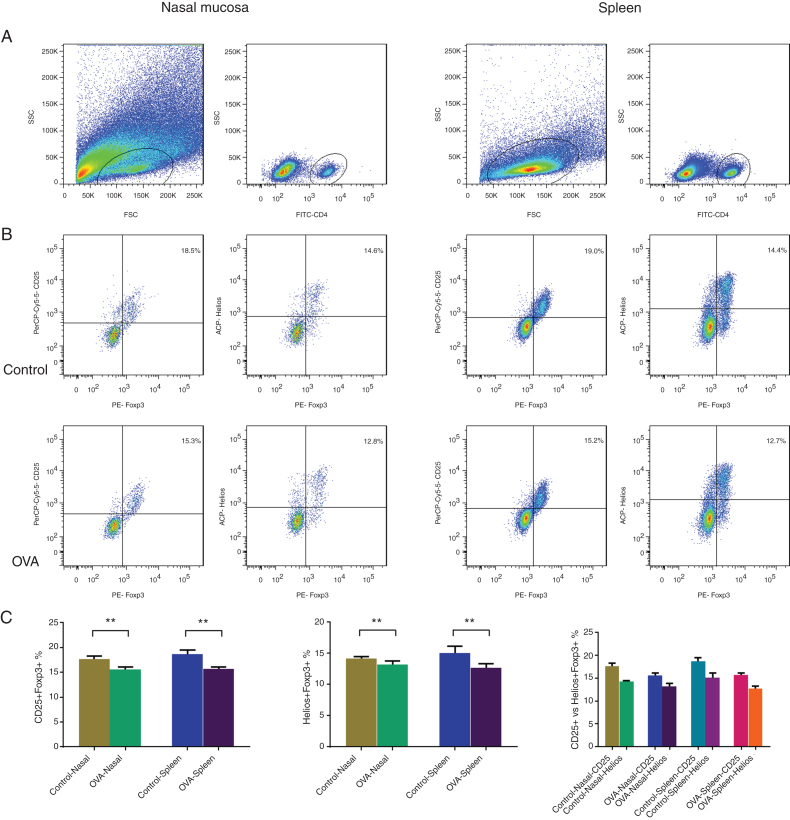
CD25^+^Tregs and Helios^+^Tregs were enumerated using flow cytometry in nasal mucosa and spleen of an AR murine model. Gating of CD4^+^ T cell populations (A). Identifying CD25^+^Tregs and Helios^+^Tregs in the control and OVA groups. The statistics are shown in (C). **p < 0.01.

The nasal mucosa and spleens of five AR mice and five control mice were examined immunohistochemically using CD4, CD25, and Foxp3 fluorescent antibodies. CD25^+^Tregs in CD4^+^cells were analyzed using flow cytometry (Fig. 2A–B) and were significantly less abundant in the nasal mucosa and spleens of AR mice (Fig. 2C).

### Fewer Helios^+^Tregs in nasal mucosa and spleens of the AR model

Coexpression of Foxp3 with Helios is thought to represent a key functional phase of Treg cells. To identify this functional group of Treg cells, nasal mucosa and spleen cells were stained with CD4, Helios, Foxp3 antibodies. The percentage of Helios^+^Tregs was analyzed using flow cytometry (Fig. 2A–B). There were fewer Helios^+^Tregs in nasal mucosa and spleens of AR model (Fig. 2C).

### Fewer Helios^+^Tregs than CD25^+^Tregs in nasal mucosa and spleens of AR model and control mice

Foxp3 coexpression was analyzed with the traditional marker CD25 and Helios in the mucosal layer of the nose and splenocytes of the AR and control mice to compare the percentages of CD25^+^Tregs and Helios^+^Tregs. In both the splenic and nasal mucosa cells of both AR and normal mice, there were fewer Helios^+^Tregs than CD25^+^Tregs (Fig. 2C). Therefore, there were fewer Tregs when using Foxp3 coexpressed with Helios than with CD25.

### Fewer Helios^+^Foxp3^+^, Helios^+^CD25^+^, and Helios^+^Foxp3^+^CD25^+^Tregs in nasal mucosa of the AR model; Helios might not be a specific marker of Treg cells

**Figure 3:**
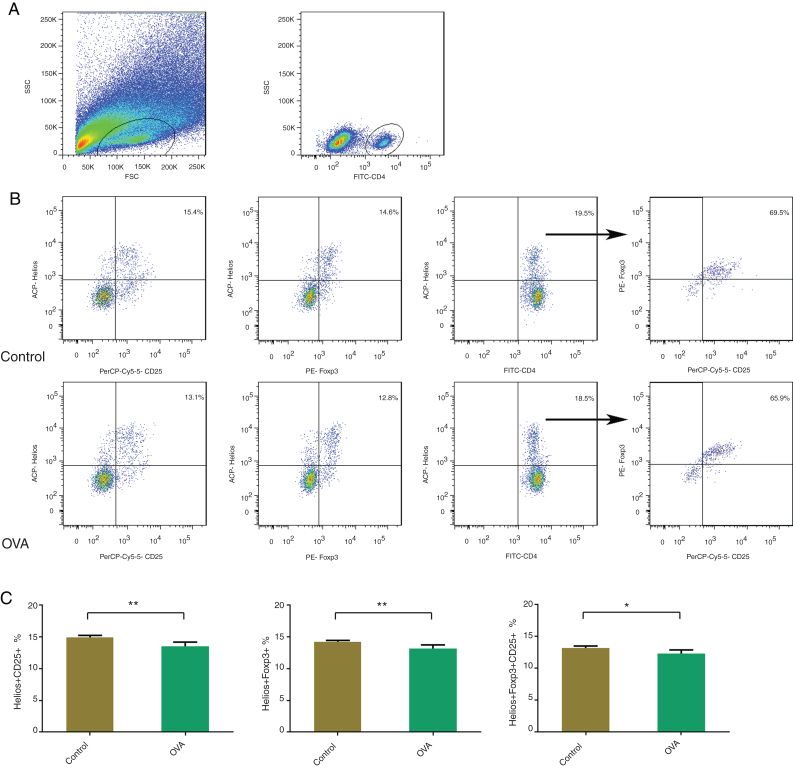
Using flow cytometry, Helios^+^Foxp3^+^, Helios^+^CD25^+^, and Helios^+^Foxp3^+^CD25^+^ were counted in nasal mucosa of an AR murine model. Gating of CD4^+^ T cell populations (A). Identifying Helios^+^Foxp3^+^, Helios^+^CD25^+^, and Helios^+^Foxp3^+^CD25^+^ in control and OVA groups. The statistics are shown in (C). **p < 0.01, *p < 0.05.

Helios is expressed in a subset of Foxp3^+^Tregs and was thought to be a marker of Treg. To compare the proportions of Helios^+^Foxp3^+^, Helios^+^CD25^+^, and Helios^+^Foxp3^+^CD25^+^ in the nasal mucosa between the control and AR mice, Helios-positive cells, CD25-positive cells, and Helios–CD25 double-positive cells were identified after CD4^+^T gating. There were fewer Helios^+^Tregs, Helios^+^CD25^+^, and Helios^+^Foxp3^+^CD25^+^Tregs in nasal mucosa of AR mice (Fig. 3). Helios was most strongly expressed in CD4^+^Foxp3^+^CD25^+^T cells, followed by CD4^+^ Foxp3^−^CD25^−^T cells, which suggests that Helios is not a specific marker of Treg cells.

**Figure 4:**
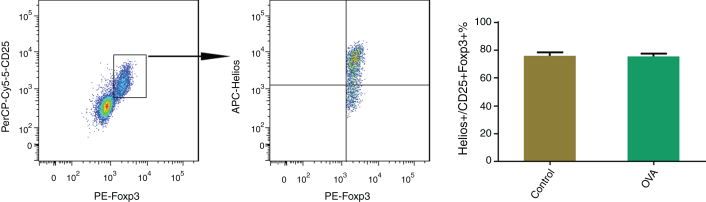
Comparison of Helios^+^in CD25^+^Tregs in spleen of the OVA and control groups. (A) The CD25^+^Tregs population was gated in CD4^+^ T cells. (B) Helios-positive cells within CD25^+^Tregs. The statistical data are shown in (C).

Approximately 75% of CD25^+^Tregs were Helios^+^ in spleens of AR and control mice. To determine the proportion of Helios^+^ in CD25^+^Tregs, the CD25^+^Tregs in spleens of AR and control mice were first identified, and then the Helios-positive cells were identified. Approximately 75% of the CD25^+^Tregs were Helios^+^ in the splenocytes of both AR and control mice (Fig. 4).

## Discussion

This is the first study to explore Helios^+^Tregs in nasal mucosa and spleen in an AR murine model. We found fewer Helios^+^Tregs in the AR group than in the controls, in both nasal mucosa cells and splenocytes. Comparing the CD25^+^Tregs and Helios^+^Tregs gating strategies, we found fewer Helios^+^Tregs than CD25^+^Tregs in both the AR and control groups, for both nasal mucosa and splenic cells.

Allergic rhinitis results from immunological crosstalk in the body, which climaxes with the deregulation of diverse inflammatory players. Tregs prevent immune responses and play the crucial role in allergic hyposensitization. Tregs inhibit effector T-cells during the Th1/Th2 phenotype inflammatory response and are therefore essential for preventing potentially harmful severe immune responses directed against foreign antigens.^13^, ^14^

Traditionally, CD25^+^Foxp3^+^ has been gated as a Treg cell. This work found fewer CD25^+^Tregs in AR mice, consistent with previous studies.^15^, ^16^, ^17^ However, studies have found that this strategy does not represent the true inhibitory function of Treg cells, even in specific T-cell induction scenarios, because CD25 and Foxp3 (two important Treg markers) are also present in activated non-Treg cells.^18^, ^19^

In 2006, Sugimoto et al. first suggested that Helios, a member of the Ikaros family of zinc-finger proteins, was a marker of natural Treg.^20^ At that time, Helios was considered to be expressed exclusively on Foxp3^+^Tregs. However, it was shown that both Helios^+^ and Helios^−^ markers exist in nTreg compartments after a few years.^21^, ^22^ Then, Helios expression was noted in 65%–75% of Treg cells and identified as a novel marker of activated nTregs, with an important suppression function; it was proposed that Helios is a marker of thymic-derived Treg.^23^, ^24^, ^25^

Foxp3 is the most specific marker of Tregs and assessment of Helios may help to identify Treg cell subsets with consistent suppressive activity.^10^, ^11^, ^23^, ^26^

In the current work, Helios expression was not restricted to CD4^+^cells, but was also seen in CD4^−^ cells (data not show). Furthermore, Helios was not expressed in Foxp3^+^Tregs, which suggested that Helios is not a specific marker of Treg cells. Therefore, gating CD4^+^Helios^+^ alone does not represent all Treg cells.

We identified Helios^+^Tregs in the nasal mucosa and spleen cells of a murine AR model and found lower levels of Helios^+^Tregs in AR compared with the control. This indicates that the suppressive activity of Tregs was inhibited in AR, leading to increased activation of T helper cells, such as Th2, and to the development of the disease.

Helios may be a useful marker for identifying bona fide Tregs under conditions that induce immune activation because, unlike CD25, its expression remains stable during T-cell activation.^22^ In the present study, the Foxp3^+^ Helios^+^ subset identified Tregs markedly less frequently than Foxp3 and CD25 coexpression, in both the control and AR groups, and around 75 % of CD25^+^Foxp3^+^ Tregs were Helios^+^.

In AR, a subgroup of Treg cells has inhibitory effects. This study showed that the use of CD25 could artificially increase the proportion of Tregs identified and may not fully reflect their inhibitory ability, whereas assessing Foxp3 and Helios coexpression is a much more precise method of identification.

The current study had several limitations. First, relatively few animals were studied. Second, this study did not consider the relationships between Treg and Th subgroups. Nevertheless, this study was informative because, as far as we know, it is the first to compare the proportions of Helios^+^Tregs and CD25^+^Tregs in nasal mucosa and spleen of a murine model of AR.

Further studies are still needed to define in detail the functional and biological importance of Helios^+^Tregs in AR, and the potential use of the marker as a therapeutic target. However, because Foxp3 and Helios are intracellular proteins, it is currently impossible to isolate living functional Helios^+^ Tregs, and to conduct suppression function assay to analyze the mechanism more fully; research is ongoing.

## Conclusion

In summary, this research is the first to identify Helios^+^ Tregs in AR. The results suggest that gating true inhibitory Tregs with the coexpression of Foxp3 and Helios might be more useful than relying on the expression of CD25. Our results provide new insight for Treg studies of AR, and potential use of the marker as a therapeutic target.

## Conflicts of interest

The authors declare no conflicts of interest.
